# Supplementary material for: Myristic acid as a checkpoint to regulate STING-dependent autophagy and interferon responses by promoting N-myristoylation
Source: Nat Commun. 2023 Feb 7;14:660. doi: 10.1038/s41467-023-36332-3 (PMC9905541; doi:10.1038/s41467-023-36332-3)

Supplementary Table 1. Sequences of polymerase chain reaction (PCR) primers used in this study.

	Prime	Sequence
β-actin	Forwar	5'-TGTTACCAACTGGGACGACA-3'
	Revers	5'-CTGGGTCATCTTTTCACGGT- 3'
Ifnb1	Forwar	5'-ATGAGTGGTGGTTGCAGGC- 3'
	Revers	5'-TGACCTTTCAAATGCAGTAGATTCA-3'
Cxcl10	Forwar	5'-ATCATCCCTGCGAGCCTATCCT-3'
	Revers	5'-GACCTTTTTTGGCTAAACGCTTTC-3'
Ifit1	Forwar	5′-TGCTGAGATGGACTGTGAGGAA-3′
	Revers	5′-TCTTGGCGATAGGCTACGACTG-3′
Ifit2	Forwar	5′-CCTAAACAGTTACTCCACCTTCG-3′
	Revers	5′-TTGCTGACCTCCTCCATTCT-3′
Isg15	Forwar	5′-AGAAGCAGATTGCCCAGAAG-3′
	Revers	5′-TGCGTCAGAAAGACCTCATAGA-3′
Tnfa	Forwar	5′-GCCACCACGCTCTTCTGTCT-3′
	Revers	5′-TGAGGGTCTGGGCCATAGAAC-3′
Il6	Forwar	5′-ACAACCACGGCCTTCCCTAC-3′
	Revers	5′-CATTTCCACGATTTCCCAGA-3′
UL30	Forwar	5′-CATCACCGACCCGGAGAGGGAC-3′
	Revers	5′-GGGCCAGGCGCTTGTTGGTGTA-3′
Nmt1	Forwar	5′-GAGTGGTGTTGCCAAAGCCTGT-3′
	Revers	5′-CAGTCGGTAGAGCTTCATGGTG-3′
Nmt2	Forwar	5′-GTGCCATTCCAGCAAACATCCG-3′
	Revers	5′-CAGCCTGAAAGATGCCTTCCAG-3′

hIFNB	*Forwar*	*5′-CAACAAGTGTCTCCTCCAAAT-3′*
	d	*5′-TCTCCTCAGGGATGTCAAAG-3′*
hβ-actin	*Forwar*	*5′-GGAAATCGTGCGTGACATTAA-3′*

Supplementary Table 2. List of abbreviations.

Abbreviations	
STING	Stimulator-of-interferon gene
IFN	Interferon
cGAS	Cyclic GMP-AMP synthase
TBK1	TANK-binding kinase 1
IRF3	Interferon regulatory factor 3
MyD88	Myeloid differentiation factor 88
TLR	Toll-like receptor
NMT	N-myristoyltransferase
AMPK	AMP-activated protein kinase
TRAM	TRIF-related adaptor molecule
ARF1	ADP-ribosylation factor 1
COP-II	Coat protein complex II
DHCR7	7-dehydrocholesterol reductase
ISG	IFN-stimulated gene
HSV-1	Herpes simplex virus-1
ISD	Interferon-stimulating DNA
SeV	Sendai virus
cGAMP	Cyclic guanosine monophosphate-adenosine monophosphate
DMXAA	5,6-dimethylxanthenone-4-acetic acid
ER	Endoplasmic reticulum
ERGIC	ER-Golgi intermediate compartment
SFA	Saturated fatty acid
FAS	Fatty acid synthesis

PM	Peritoneal macrophages
MEF	Mouse embryonic fibroblast
qPCR	Quantitative polymerase chain reaction
ELISA	Enzyme-linked immunosorbent assay

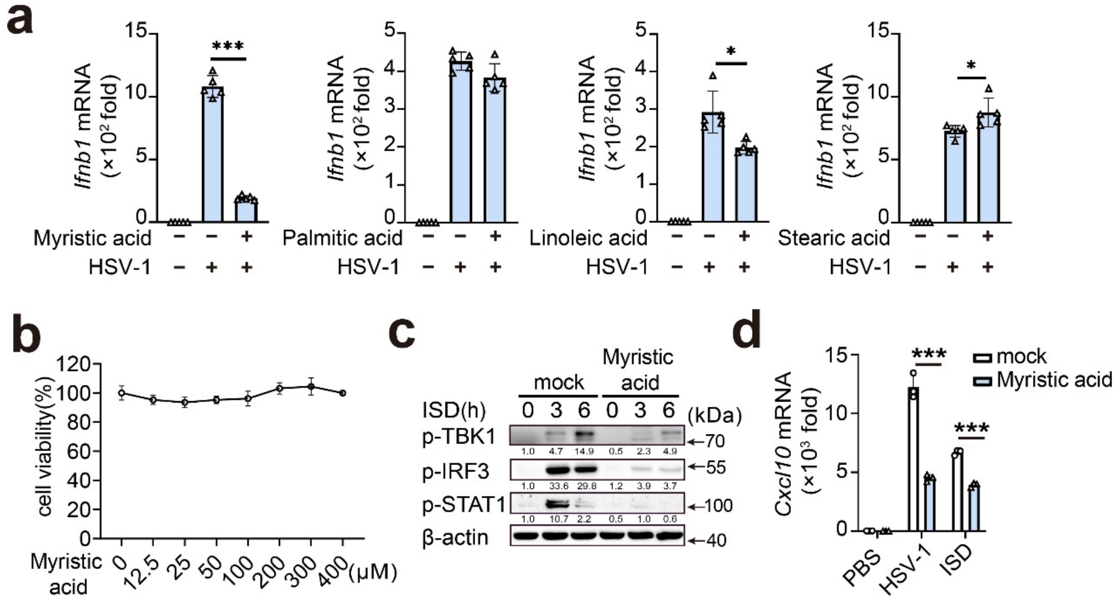

Supplementary Figure 1. Myristic acid suppresses cGAS-dependent antiviral immune responses. a, qPCR analysis of *Ifnb1* mRNA in peritoneal macrophages (PMs) treated with the indicated saturated fatty acid (SFA) (50 μM), and then infected with herpes simplex virus 1 (HSV-1), n=5 samples examined over 3 independent experiments. **b,** Cell viability analysis of PMs treated with increasing concentrations of myristic acid, n=3 samples examined over 3 independent experiments. **c,** Immunoblot analysis of p-TBK1, p-IRF3, and p-STAT1 in PMs pretreated with solvent (mock) or myristic acid, and then transfected with interferon (IFN)-stimulating DNA (ISD). **d,** Quantitative polymerase chain reaction (qPCR) analysis of *Cxcl10* mRNA in PMs pretreated with solvent (mock) or myristic acid, and then infected with HSV-1 or stimulated with ISD, n=3 samples examined over 3 independent experiments. Statistical significance was determined by unpaired two-sided multiple Student's t-tests in a and d. Data are shown as mean ± standard deviation (SD) or typical photographs and are representative of three biological independent experiments with similar results. *** $P < 0.001$. Source data is provided in the Source Data File.

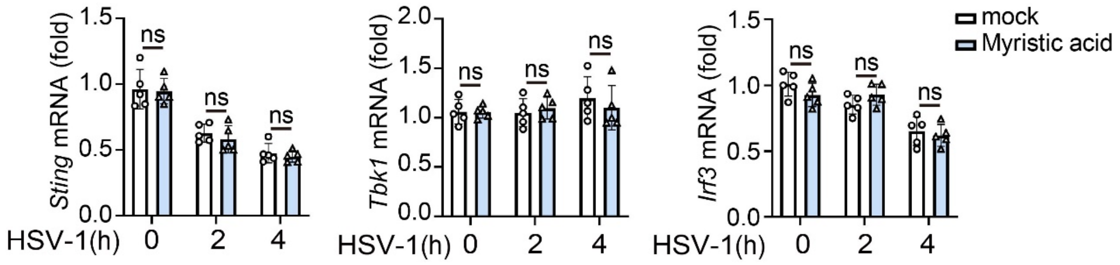

Supplementary Figure 2. Myristic acid has no effects on mRNA expression of *Sting*, *Tbk1*, and *Irf3*. Quantitative polymerase chain reaction (qPCR) analysis of *Sting*, *Tbk1*, and *Irf3* mRNA in peritoneal macrophages (PMs) treated with solvent (mock) or myristic acid, and then infected with herpes simplex virus 1 (HSV-1), n=5 samples examined over 3 independent experiments. Statistical significance was determined by unpaired two-sided multiple Student's t-tests. Data are shown as mean ± standard deviation (SD) and are representative of three biological independent experiments with similar results, ns, not significant ($P > 0.05$). Source data is provided in the Source Data File.

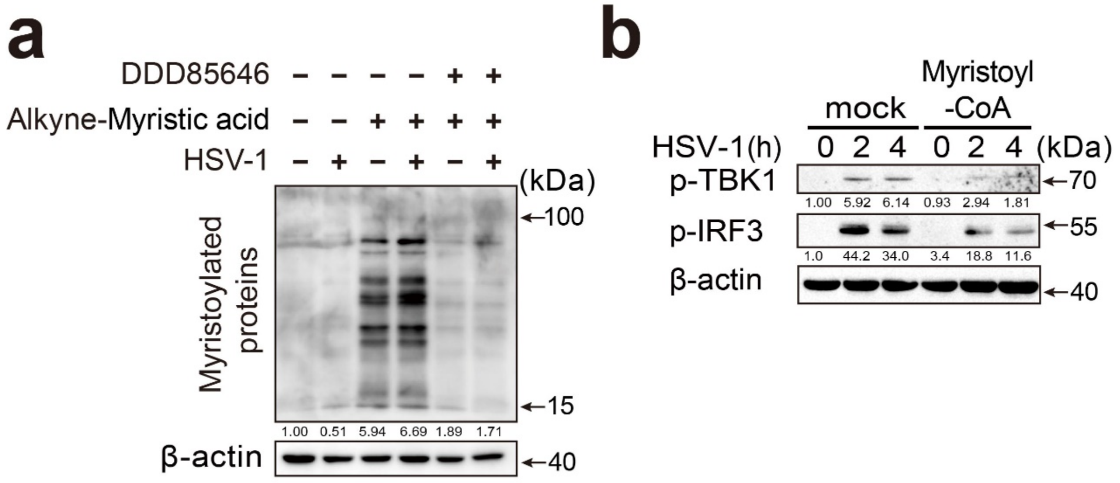

Supplementary Figure 3. Myristic acid enhances N-myristoylation and attenuates cGAS signaling. a, Immunoblot analysis of total N-myristoylated proteins by selective labeling with alkyne-myristic acid in herpes simplex virus 1 (HSV-1)-infected peritoneal macrophages (PMs). Typical photographs of one representative from three independent experiments. **b,** Immunoblot analysis of indicated proteins in PMs treated with solvent (mock) or myristoyl-CoA, followed by HSV-1 infection. Data are shown as typical photographs and are representative of three biological independent experiments with similar results.

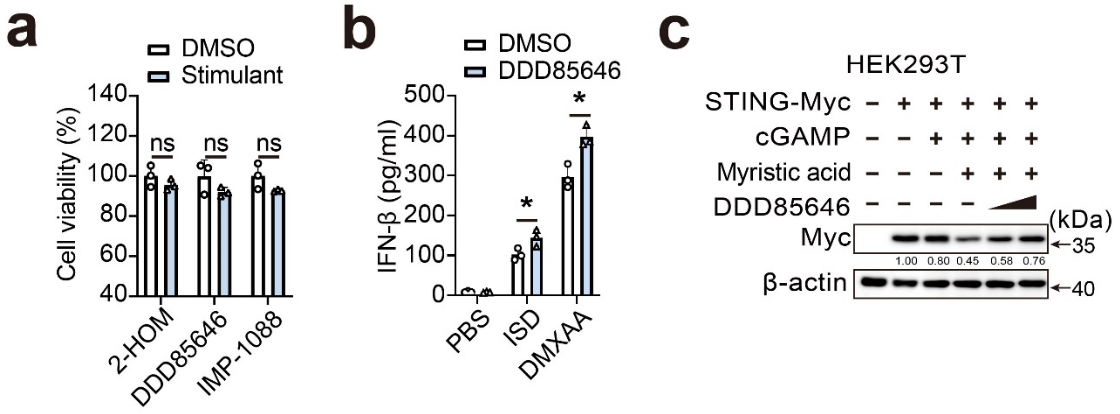

Supplementary Figure 4. Myristic acid suppresses cGAS-STING pathway via facilitating myristoylation. a, Cell viability analysis of peritoneal macrophages (PMs) treated with indicated stimulants, n=3 samples examined over 3 independent experiments. **b,** Enzyme-linked immunosorbent assay (ELISA) analysis of interferon (IFN)-β secretion in PMs pretreated with DMSO or DDD85646, following stimulation with ISD or DMXAA, n=3 samples examined over 3 independent experiments. **c,** Immunoblot analysis of STING expression in STING-Myc transfected HEK293T cells pretreated with DDD85646 and myristic acid, following cGAMP stimulation. Statistical significance was determined by unpaired two-sided multiple Student's t-tests in a and b. Data are shown as mean ± standard deviation (SD) or typical photographs and are representative of three biological independent experiments with similar results. ns, not significant ($P > 0.05$), * $P < 0.05$. Source data is provided in the Source Data File.

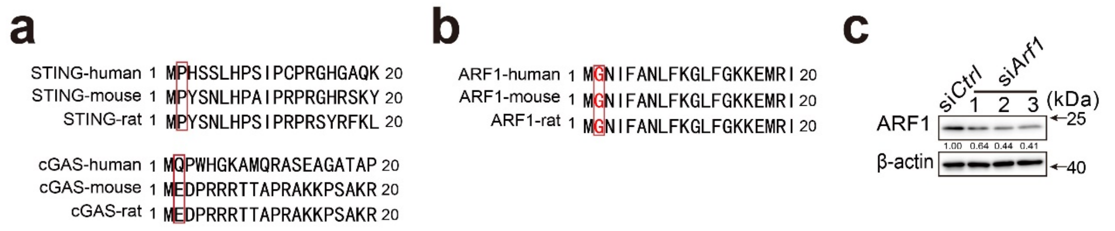

Supplementary Figure 5. Alignment of STING, cGAS, and ARF1. a-b, A conserved N-terminal Gly residue of adenosine diphosphate (ADP)-ribosylation factor 1 (ARF1) in three species is marked as red. **c,** Immunoblot analysis of ARF1 expression in peritoneal macrophages (PMs) treated transfected with negative control siRNA (si*Ctrl*) or *Arf1* siRNA (si*Arf1*) for 48 h. Data are shown as typical photographs and are representative of three biological independent experiments with similar results.

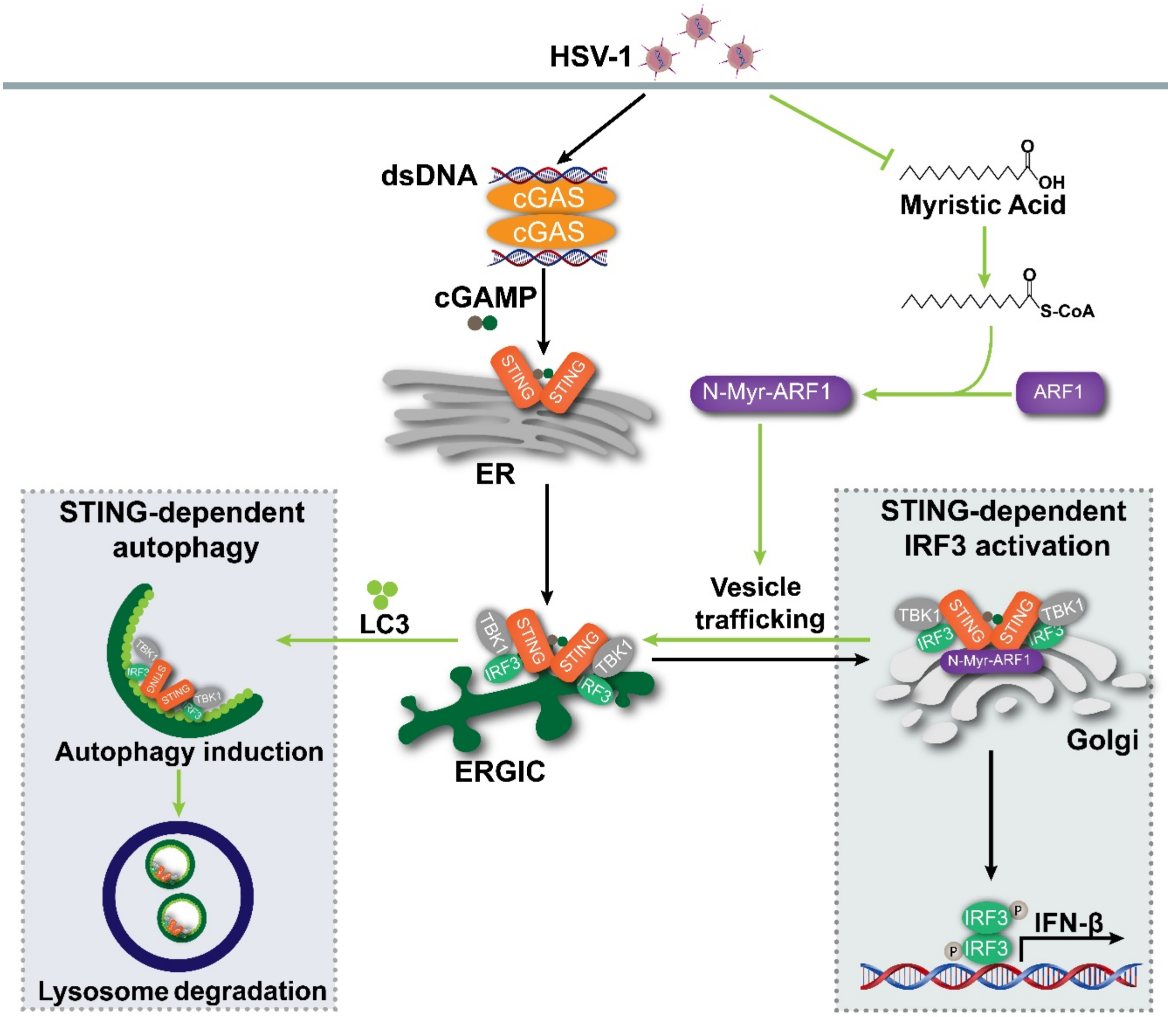

Supplementary Figure 6. Schematic representation of the role of myristic acid in balancing STING-dependent autophagy and interferon (IFN) responses. Myristic acid enhances N-myristoylation of adenosine diphosphate (ADP)-ribosylation factor 1 (ARF1), a key modification required for its function in directing STING membrane trafficking. Consequently, myristic acid facilitates STING trafficking from the Golgi to endoplasmic reticulum (ER)–Golgi intermediate compartment (ERGIC), a key step for its autophagy degradation, and then suppresses STING-dependent IFN responses. Herpes simplex virus 1 (HSV-1) hijacks STING-dependent autophagy by downregulating myristic acid, while the reduction of myristic acid in macrophages enhances STING-dependent IFN responses.

Unprocessed Scans

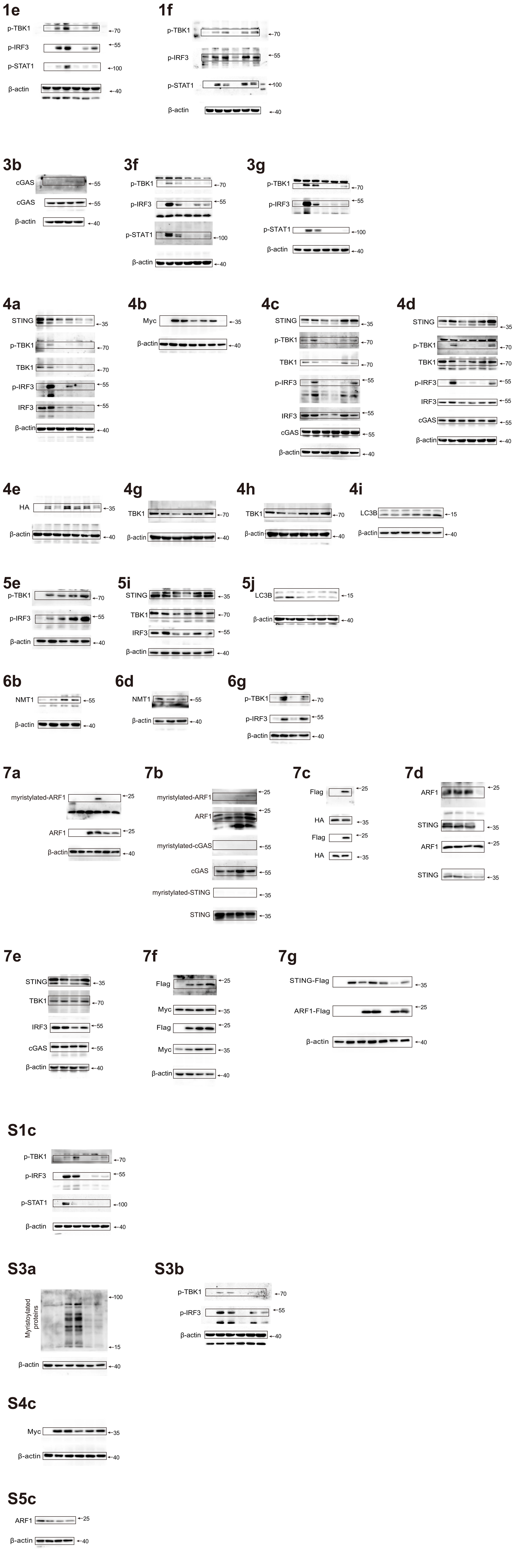

Supplement: Supplementary file 1 — Supplementary Information [file 41467_2023_36332_MOESM1_ESM.pdf]
