# Supplementary material for: Myristic acid as a checkpoint to regulate STING-dependent autophagy and interferon responses by promoting N-myristoylation
Source: Nat Commun. 2023 Feb 7;14:660. doi: 10.1038/s41467-023-36332-3 (PMC9905541; doi:10.1038/s41467-023-36332-3)
Supplement: Supplementary file 3 — Reporting Summary [file 41467_2023_36332_MOESM3_ESM.pdf]

nature portfolio

Corresponding author(s): Wei Zhao

Last updated by author(s): Dec 26, 2022

Reporting Summary

Nature Portfolio wishes to improve the reproducibility of the work that we publish. This form provides structure for consistency and transparency in reporting. For further information on Nature Portfolio policies, see our Editorial Policies and the Editorial Policy Checklist.

Statistics

For all statistical analyses, confirm that the following items are present in the figure legend, table legend, main text, or Methods section.

n/a	Confirmed	
☐	☒	The exact sample size (*n*) for each experimental group/condition, given as a discrete number and unit of measurement
☐	☒	A statement on whether measurements were taken from distinct samples or whether the same sample was measured repeatedly
☐	☒	The statistical test(s) used AND whether they are one- or two-sided<br>*Only common tests should be described solely by name; describe more complex techniques in the Methods section.*
☒	☐	A description of all covariates tested
☒	☐	A description of any assumptions or corrections, such as tests of normality and adjustment for multiple comparisons
☐	☒	A full description of the statistical parameters including central tendency (e.g. means) or other basic estimates (e.g. regression coefficient) AND variation (e.g. standard deviation) or associated estimates of uncertainty (e.g. confidence intervals)
☐	☒	For null hypothesis testing, the test statistic (e.g. *F*, *t*, *r*) with confidence intervals, effect sizes, degrees of freedom and *P* value noted<br>*Give P values as exact values whenever suitable.*
☒	☐	For Bayesian analysis, information on the choice of priors and Markov chain Monte Carlo settings
☒	☐	For hierarchical and complex designs, identification of the appropriate level for tests and full reporting of outcomes
☒	☐	Estimates of effect sizes (e.g. Cohen's *d*, Pearson's *r*), indicating how they were calculated

Our web collection on statistics for biologists contains articles on many of the points above.

Software and code

Policy information about availability of computer code

Data collection	Amersham Imager 680 (GE Healthcare Life Sciences,USA), GeneGnome XR (Synoptics Ltd. Synoptics Ltd, England), Infinite M200 Pro (Tecan,Switzerland), LSM 880 with Airyscan (Zeiss, Germany), CFX Connect(Bio-Rad,USA), Agilent7890B-7000D(Agilent, USA).
Data analysis	Prism 8(GraphPad,California,USA), ImageQuant TL 8.0 (GE Healthcare Life Sciences,USA), ZEN blue and gray edition 3.1 (Zeiss, Germany), ImageJ v1.53v(National Institutes of Health, USA), ChemStation C.01.09(Agilent,USA).

For manuscripts utilizing custom algorithms or software that are central to the research but not yet described in published literature, software must be made available to editors and reviewers. We strongly encourage code deposition in a community repository (e.g. GitHub). See the Nature Portfolio guidelines for submitting code & software for further information.

Data

Policy information about availability of data

All manuscripts must include a data availability statement. This statement should provide the following information, where applicable:

- Accession codes, unique identifiers, or web links for publicly available datasets
- A description of any restrictions on data availability
- For clinical datasets or third party data, please ensure that the statement adheres to our policy

All data supporting the findings of this study are available within the article and the data generated in this study are provided in the Supplementary Information and Source Data file.

Field-specific reporting

Please select the one below that is the best fit for your research. If you are not sure, read the appropriate sections before making your selection.

- [x] Life sciences
- [ ] Behavioural & social sciences
- [ ] Ecological, evolutionary & environmental sciences

For a reference copy of the document with all sections, see nature.com/documents/nr-reporting-summary-flat.pdf

Life sciences study design

All studies must disclose on these points even when the disclosure is negative.

Sample size	Sample size for each experiment is indicated in the legend. No statistical methods were used to predetermine sample sizes. Sample size was chosen based on previous experiments and comparable(DOI: 10.1038/ncomms13727;DOI: 10.1038/s41590-020-0699-0).
Data exclusions	No exclusion of data was made.
Replication	All experimental findings were reproduced in multiple independent experiments. For experiments using mouse peritoneal macrophages, each independent experiment used mouse peritoneal macrophages isolated from another mouse. For each figure, the number of independent experiments or biological replicates is indicated in the figure legends. Western blot pictures are from a representative experiment and the number of independent repeats is clearly indicated in the figure legends. All attempts at data replication were successful.
Randomization	No statistical methods were used for randomization. For in vitro experiments, mouse peritoneal macrophages were isolated from randomly chosen wild-type mice, Sting-deficient mice or Ifnar1-deficient mice, and processing was performed simultaneously and in parallel for all conditions within each experiment. For in vivo experiments, wild-type mice were randomly allocated into experimental groups.
Blinding	The investigators were blinded during data collection and analysis where possible. For western blotting, the experiments were not blinded, due to careful experimental setup and design. For qRT-PCR, ELISA and confocal microscopy, the experiments were blinded to the conditions during the experiments or analysis.

Reporting for specific materials, systems and methods

We require information from authors about some types of materials, experimental systems and methods used in many studies. Here, indicate whether each material, system or method listed is relevant to your study. If you are not sure if a list item applies to your research, read the appropriate section before selecting a response.

Materials & experimental systems

n/a	Involved in the study	
[ ]	[x]	Antibodies
[ ]	[x]	Eukaryotic cell lines
[x]	[ ]	Palaeontology and archaeology
[ ]	[x]	Animals and other organisms
[x]	[ ]	Human research participants
[x]	[ ]	Clinical data
[x]	[ ]	Dual use research of concern

Methods

n/a	Involved in the study	
[x]	[ ]	ChIP-seq
[x]	[ ]	Flow cytometry
[x]	[ ]	MRI-based neuroimaging

Antibodies

Antibodies used	Anti-STING (1:1000, #13647), anti-TBK1 (1:1000, #3504), anti-p-IRF3 (1:1000, #4947), anti-IRF3 (1:1000, #4302), anti-p-STAT1 (1:1000, #9167), anti-cGAS (1:1000, #31659), and anti-streptavidin-HRP (1:1000, #3999) were purchased from Cell Signaling Technology. Anti-NMT1 (1:2000, ab186123), anti-ICP5 (1:3000, ab6508), anti-GM130 (1:100, ab52649), anti-LC3B (1:2000, ab192890), and anti-p-TBK1 (1:1000, ab109272) were purchased from Abcam. Anti-Myc (1:5000, M4439) and anti-Flag (1:5000, F1804) antibodies were purchased from Sigma. Anti-β-actin (1:20000, 66009-I-Ig) and anti-ARF1(1:1000, 20226-1-AP) were purchased from Proteintech. Anti-HA (1:2000, TA180128) was purchased from Origene.
Validation	Commercial available Western blot and immunofluorescence antibodies were selected based on their antigen specificity and suggested application as described on the manufacturer's website and data sheets.<br>Anti-STING (1:1000, #13647) validate for WB and IF, https://www.cellsignal.co.uk/products/primary-antibodies/sting-d2p2f-rabbit-mab/13647?N=0+102236+4294956287&Nrpp=200&No=6000&fromPage=plp;<br>Anti-TBK1 (1:1000, #3504) validate for WB, https://www.cellsignal.co.uk/products/primary-antibodies/tbk1-nak-d1b4-rabbit-mab/3504?site-search-type=Products&N=4294956287&Ntt=%233504&fromPage=plp&_requestid=4637060;<br>Anti-p-IRF3 (1:1000, #4947) validate for WB, https://www.cellsignal.co.uk/products/primary-antibodies/phospho-irf-3-ser396-4d4g-rabbit-mab/4947?site-search-type=Products&N=4294956287&Ntt=%234947&fromPage=plp&_requestid=4637183;<br>Anti-IRF3 (1:1000, #4302) validate for WB, https://www.cellsignal.co.uk/products/primary-antibodies/irf-3-d83b9-rabbit-mab/4302?site-search-type=Products&N=4294956287&Ntt=+%234302&fromPage=plp&_requestid=4637282;

Anti-p-STAT1 (1:1000, #9167) validate for WB, https://www.cellsignal.co.uk/products/primary-antibodies/irf-3-d83b9-rabbit-mab/4302?site-search-type=Products&N=4294956287&Ntt=+%234302&fromPage=plp&_requestid=4637282;
Anti-cGAS (1:1000, #31659) validate for WB, https://www.cellsignal.co.uk/products/primary-antibodies/cgas-d3o8o-rabbit-mab-mouse-specific/31659?site-search-type=Products&N=4294956287&Ntt=%2331659&fromPage=plp&_requestid=4637537;
Anti-NMT1 (1:2000, ab186123) validate for WB, https://www.abcam.cn/nmt1nmt-antibody-ab186123.html;
Anti-ICP5 (1:3000, ab6508) validate for WB and IHC, https://www.abcam.cn/hsv1--hsv2-icp5-major-capsid-protein-antibody-3b6-bsa-and-azide-free-ab6508.html;
Anti-GM130 (1:100, ab52649) validate for IF, https://www.abcam.cn/gm130-antibody-ep892y-cis-golgi-marker-ab52649.html;
Anti-LC3B (1:2000, ab192890) validate for WB and IF, https://www.abcam.cn/lc3b-antibody-epr18709-autophagosome-marker-ab192890.html;
Anti-p-TBK1 (1:1000, ab109272) validate for WB, https://www.abcam.cn/naktbk1-phospho-s172-antibody-epr28672-ab109272.html;
Anti-Myc (1:5000, M4439) validate for WB and IP, https://www.sigmaaldrich.cn/CN/zh/product/sigma/m4439;
Anti-Flag (1:5000, F1804) validate for WB and IP, https://www.sigmaaldrich.cn/CN/zh/product/sigma/f1804;
Anti-β-actin (1:20000, 66009-I-Ig) validate for WB, https://www.ptgcn.com/products/Pan-Actin-Antibody-66009-1-Ig.htm;
Anti-ARF1(1:1000, 20226-1-AP) validate for WB, https://www.ptgcn.com/products/ARF1-Specific-Antibody-20226-1-AP.htm;
Anti-HA (1:2000, TA180128) validate for WB and IP, https://www.origene.com/catalog/antibodies/tag-antibodies/ta180128/ha-mouse-monoclonal-antibody-clone-cb051.

Eukaryotic cell lines

Policy information about cell lines

Cell line source(s)	HEK293T cells, THP-1 and Bj cells were from ATCC. HEK293T-STING A162 were from InvivoGen.
Authentication	All used cells were authenticated by morphology, karyotyping, and polymerase chain reaction (PCR)-based approaches.
Mycoplasma contamination	The MycoProbe detection kit (R&D systems) was used to check for mycoplasma contamination, and all cell lines were tested negative for mycoplasma contamination.
Commonly misidentified lines (See ICLAC register)	No commonly misidentified cell lines were used in the study.

Animals and other organisms

Policy information about studies involving animals; ARRIVE guidelines recommended for reporting animal research

Laboratory animals	Sting-deficient mice (025805) and Ifnar1-deficient mice (028288) were obtained from the Jackson Laboratory. C57BL/6 mice were from Vital River Laboratory Animal Technology Co. (Beijing,China). All animals were used at 6 to 11 weeks of age. Both male and female mice were used. All mice were maintained on a 12 hours light/dark cycle with constant ambient temperature (22 –24 °C) and humidity (~60%).
Wild animals	No wild animals used.
Field-collected samples	No field-collected samples used.
Ethics oversight	All animal experiments ethically conducted according to the criteria of the National Institute of Health Guide for the Care and Use of Laboratory Animals and approved by the Scientific Investigation Board of the School of Basic Medical Science, Shandong University, Jinan, Shandong Province, China.

Note that full information on the approval of the study protocol must also be provided in the manuscript.
